# Activation of STING signaling aggravates chronic alcohol exposure‐induced cognitive impairment by increasing neuroinflammation and mitochondrial apoptosis

**DOI:** 10.1111/cns.14689

**Published:** 2024-03-22

**Authors:** Xinrou Lin, Xiangpen Li, Chenguang Li, Hongxuan Wang, Lubin Zou, Jingrui Pan, Xiaoni Zhang, Lei He, Xiaoming Rong, Ying Peng

**Affiliations:** ^1^ Department of Neurology, Sun Yat‐Sen Memorial Hospital Sun Yat‐Sen University Guangzhou China; ^2^ Nanhai Translational Innovation Center of Precision Immunology Sun Yat‐Sen Memorial Hospital Foshan China; ^3^ Shenshan Medical Center, Sun Yat‐sen Memorial Hospital Sun Yat‐sen University Shanwei China; ^4^ Guangdong Provincial Key Laboratory of Malignant Tumor Epigenetics and Gene Regulation, Sun Yat‐Sen Memorial Hospital Sun Yat‐Sen University Guangzhou China

**Keywords:** alcohol use disorder, apoptosis, cognitive impairment, neuroinflammation, STING

## Abstract

**Aims:**

Chronic alcohol exposure leads to persistent neurological disorders, which are mainly attributed to neuroinflammation and apoptosis. Stimulator of IFN genes (STING) is essential in the cytosolic DNA sensing pathway and is involved in inflammation and cellular death processes. This study was to examine the expression pattern and biological functions of STING signaling in alcohol use disorder (AUD).

**Methods:**

Cell‐free DNA was extracted from human and mouse plasma. C57BL/6J mice were given alcohol by gavage for 28 days, and behavior tests were used to determine their mood and cognition. Cultured cells were treated with ethanol for 24 hours. The STING agonist DMXAA, STING inhibitor C‐176, and STING‐siRNA were used to intervene the STING. qPCR, western blot, and immunofluorescence staining were used to assess STING signaling, inflammation, and apoptosis.

**Results:**

Circulating cell‐free mitochondrial DNA (mtDNA) was increased in individuals with AUD and mice chronically exposed to alcohol. Upregulation of STING signaling under alcohol exposure led to inflammatory responses in BV2 cells and mitochondrial apoptosis in PC12 cells. DMXAA exacerbated alcohol‐induced cognitive impairment and increased the activation of microglia, neuroinflammation, and apoptosis in the medial prefrontal cortex (mPFC), while C‐176 exerted neuroprotection.

**Conclusion:**

Activation of STING signaling played an essential role in alcohol‐induced inflammation and mitochondrial apoptosis in the mPFC. This study identifies STING as a promising therapeutic target for AUD.

## Graphical abstract

Compared to healthy controls, individuals with alcohol use disorder (AUD) and mice chronically exposed to alcohol had increased circulating cell‐free mtDNA, which could be a potential biomarker for AUD. Activation of the STING caused inflammation in microglia and mitochondrial apoptosis in neurons. The STING agonist DMXAA aggravated alcohol‐induced cognitive impairment with upregulation of microglial activation, neuroinflammation, and apoptosis, while the STING inhibitor C‐176 exerted neuroprotection.

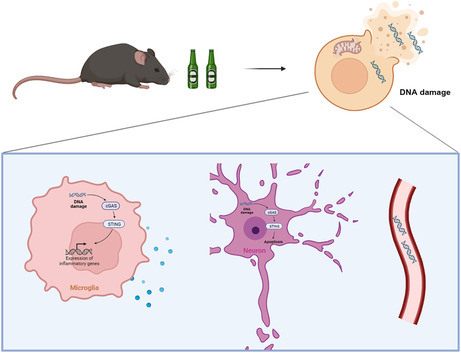

## 1 INTRODUCTION

Double‐stranded DNA (dsDNA) is sequestered within the nucleus or mitochondria. Upon gaining access to the cytosol, dsDNA that is derived from pathogenic sources or damaged cells can bind with cyclic GMP‐AMP synthase (cGAS).^1^, ^2^ Activated cGAS produces cyclic guanosine monophosphate–adenosine monophosphate (cGAMP), which serves as a second messenger detected by stimulator of interferon genes (STING), an endoplasmic reticulum‐resident protein.^1^, ^3^, ^4^ STING subsequently forms a multimer and translocates to the Golgi apparatus and promotes the phosphorylation of TANK‐binding kinase 1 (TBK1). TBK1 in turn activates interferon regulatory factor 3 (IRF3) and nuclear factor (NF)‐κB. Activated IRF3 and NF‐κB enter the nucleus and stimulate the production of proinflammatory cytokines.^5^ Apart from inflammatory responses, emerging studies suggest that activation of the STING is involved in various forms of cellular death processes, such as autophagy, ferroptosis, apoptosis, pyroptosis, and necroptosis.^6^, ^7^, ^8^ Activation of the STING signaling pathway has been implicated in pathological changes associated with autoimmune diseases, infections, and CNS diseases.^9^, ^10^, ^11^, ^12^

Alcohol (ethanol, EtOH) is one of the most commonly consumed addictive substances worldwide and alcohol use disorder (AUD) is increasingly emerging as a significant global issue of concern.^13^ Prolonged alcohol exposure can lead to various neurological disorders, including anxiety, depression, and cognitive impairment.^14^, ^15^, ^16^ The medial prefrontal cortex (mPFC), which is responsible for spatial working memory, is particularly susceptible to the detrimental effects of chronic alcohol consumption.^17^, ^18^ Preclinical studies have demonstrated that binge drinking and heavy alcohol consumption during adolescence can cause shrinkage of both gray and white matter.^18^, ^19^ Mice chronically exposed to alcohol during adolescence also exhibit persistent structural alterations in the mPFC.^20^, ^21^ Neuroinflammation and neurodegeneration have been identified as two primary mechanisms underlying chronic alcohol‐induced cognitive impairment.^22^, ^23^, ^24^

Alcohol‐induced DNA damage is one of the mechanisms underlying alcohol‐induced brain cells injury.^25^, ^26^ Toll‐like receptors (TLRs), RIG‐I‐like receptors (RLRs), and cGAS‐STING signaling are nucleic acid sensors that are critical in innate immunity.^27^ TLR4 deficiency has been shown to alleviate alcohol‐induced neuroinflammation and apoptosis in the cerebral cortex of mice.^28^ TLR2, TLR3, and TLR7 have also been implicated in alcohol‐induced inflammatory responses.^29^, ^30^, ^31^ However, it is unclear whether the STING signaling pathway is associated with alcohol‐induced brain damage. In this study, we found that individuals with AUD had higher levels of cell‐free mtDNA in plasma than healthy controls. Elevated cell‐free mtDNA in plasma was also observed in mice chronically exposed to alcohol. The STING was activated in brain cells following alcohol exposure, and its activation led to inflammatory responses and mitochondrial apoptosis. A STING agonist called DMXAA worsened alcohol‐induced cognitive impairment with modulation of microglial activation, neuroinflammation, and apoptosis within the mPFC, while a STING inhibitor called C‐176 exhibited neuroprotection. Our study reveals circulating cell‐free mtDNA as a potential biomarker underlying AUD and identifies STING as a novel target for therapeutic intervention.

## 2 MATERIALS AND METHODS

### 2.1 Animals

Male C57BL/6 mice, aged six to eight weeks and weighing 18–22 g, were obtained from Zhuhai BesTest Bio‐Tech Co., Ltd. All animal experiments were approved by the Animal Ethical and Welfare Committee of Sun Yat‐Sen Memorial Hospital, Sun Yat‐sen University. A chronic alcohol exposure model was constructed based on our previous studies with a slight modification.^32^, ^33^ In experiment 1, mice were randomly divided into three groups: the Sham group, EtOH group (25% ethanol w/v, administered i.g., 28 d, 5 g/kg/day), and EtOH + DMXAA (intraperitoneal injection, 25 mg/kg, S1537, Selleck) group. In experiment 2, mice were randomly divided into three groups: the Sham group, EtOH group (25% ethanol v/v, administered i.g., 28 d, 5 g/kg/day), and EtOH + C‐176 (intraperitoneal injection, 10 mg/kg, HY‐112906, MCE) group. DMXAA is an activator of STING and C‐176 is an inhibitor of STING. The doses of DMXAA and C‐176 were chosen based on previous studies.^34^, ^35^ DMXAA and C‐176 were diluted with a solvent consisting of 10% dimethyl sulfoxide (DMSO), 40% PEG‐300, 5% Tween‐80, and 45% sterile water. The Sham group and the EtOH group were administered the solvent alone.

### 2.2 Cell culture and cell transfection

The mouse microglial cell line BV2 and the mouse neuronal cell line PC12, purchased from Procell Life, China, were cultured in DMEM (Gibco, USA) supplemented with 10% fetal bovine serum (FBS, Excellbio, China) and 1% penicillin and streptomycin (Gibco, USA) at 37°C and 5% CO2. According to the manufacturer's instructions, BV2 cells were transfected with negative control‐siRNA (siNC) or STING‐siRNA (siSTING) (IGEBio Co., Ltd, Guangzhou, China) by Lipofectamine RNAiMAX (Thermo Fisher, USA). The concentration of siRNA used for the experiment was 50 nM or 100 nM, and the sequence of STING‐siRNA was as follows: GAGCTTGACTCCAGCGGAA.

### 2.3 Quantification of circulating cell‐free mtDNA levels

Circulating cell‐free mitochondrial DNA (cf‐mtDNA) and cell‐free nuclear DNA (cf‐nDNA) were extracted from 300 μL of plasma from humans and mice using the Plasma Free DNA Extraction Kit with magnetic beads (Tiangen, DP709). qPCR was conducted to measure the levels of human nuclear genomic genes such as glyceraldehyde‐3‐phosphate dehydrogenase (GAPDH) and telomerase reverse transcriptase (TERT) and human mitochondrial genes such as mitochondrially encoded NADH dehydrogenase 6 (MT‐ND6) and mitochondrially encoded NADH dehydrogenase 1 (MT‐ND1). In mice, the levels of nuclear DNA encoding 18S ribosomal RNA and mitochondrial genes, including cytochrome *c* oxidase 1 (MT‐COI) and the D‐loop region (MT‐Dloop), were analyzed. The cf‐mtDNA/cf‐nDNA ratios including MT‐ND1/GAPDH, MT‐ND1/TERT, MT‐ND6/GAPDH, MT‐ND6/TERT, MT‐COI/18S, and MT‐Dloop/18S were calculated. The sequences of primers for the target genes are shown in Table S1. The demographics and clinical characteristics of healthy controls and individuals with AUD included in the current study are provided in Table S2.

### 2.4 Statistical analysis

All data were presented as the means ± standard errors of the means (SEMs) and analyzed using IBM SPSS 26.0 software. Shapiro–Wilk or Kolmogorov–Smirnov normality test was used to assess data distribution. For normally distributed data, two‐tailed Student's t test was used to compare the means of two groups, and one‐way analysis of variance (ANOVA) followed by LSD for post hoc comparisons was used to compare the means of multiple groups. For data that do not exhibit a normal distribution, Mann–Whitney *U* test and Kruskal–Wallis tests were used. The level of significance was set at *p* < 0.05.

The additional material and methods were provided in the Supplemental information.

## 3 RESULTS

### 3.1 Circulating cell‐free mtDNA levels are increased in patients with AUD and mice chronically exposed to alcohol

**FIGURE 1:**
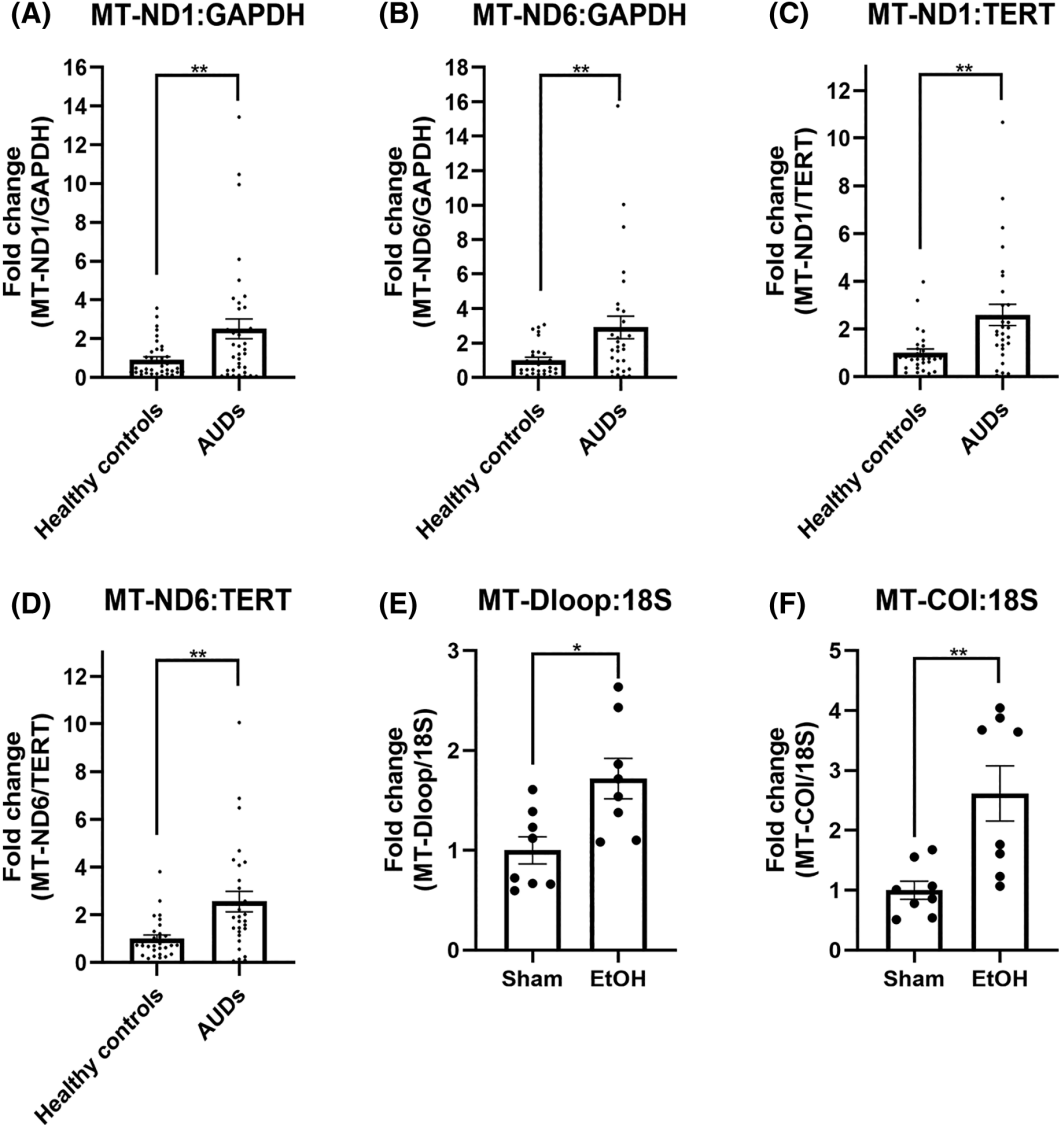
Evaluation of circulating cell‐free mtDNA levels in plasma from humans and mice. (A) Ratio of circulating cell‐free MT‐ND1 to cell‐free GAPDH in plasma from healthy controls and patients with AUD; *p* = 0.004. (B) Ratio of circulating cell‐free MT‐ND6 to cell‐free GAPDH in plasma from healthy controls and patients with AUD; *p* = 0.007. (C) Ratio of circulating cell‐free MT‐ND1 to cell‐free TERT in plasma from healthy controls and patients with AUD; *p* = 0.001. (D) Ratio of circulating cell‐free MT‐ND6 to cell‐free TERT in plasma from healthy controls and patients with AUD; *p* = 0.001. (E) Ratio of circulating cell‐free MT‐COI to cell‐free 18S in plasma from mice in the sham group and the chronic alcohol exposure group; *p* = 0.005. (F) Ratio of circulating cell‐free MT‐Dloop to cell‐free 18S in plasma from mice in the sham group and the chronic alcohol exposure group; *p* = 0.011. Healthy controls, *n* = 29; AUD patients, *n* = 29; Sham, *n* = 8; EtOH, *n* = 8. The data were analyzed using Student's *t* test and are expressed as the mean ± SEM; *p < 0.05, **p < 0.01, ***p < 0.001; ns, nonsignificant.

To investigate whether alcohol‐induced cell damage leads to increased release of dsDNA into the extracellular space, we measured the cell‐free mtDNA in the plasma of individuals with AUD (*n* = 29) and healthy controls (*n* = 29) by using qPCR. The findings revealed that the cf‐mtDNA/cf‐nDNA ratios of all four sets of reference genes in individuals with AUD were significantly higher than those in healthy controls (Figure 1A–D). Additionally, the mitochondrial gene fragment (*MT‐COI* and *MT‐Dloop*) to nuclear gene fragment (*18S*) ratios were increased in alcohol‐exposed mice (*n* = 8) in comparison to the controls (*n* = 8) (Figure 1E,F).

### 3.2 Increased STING signaling activity in the mPFC of mice chronically exposed to alcohol

**FIGURE 2:**
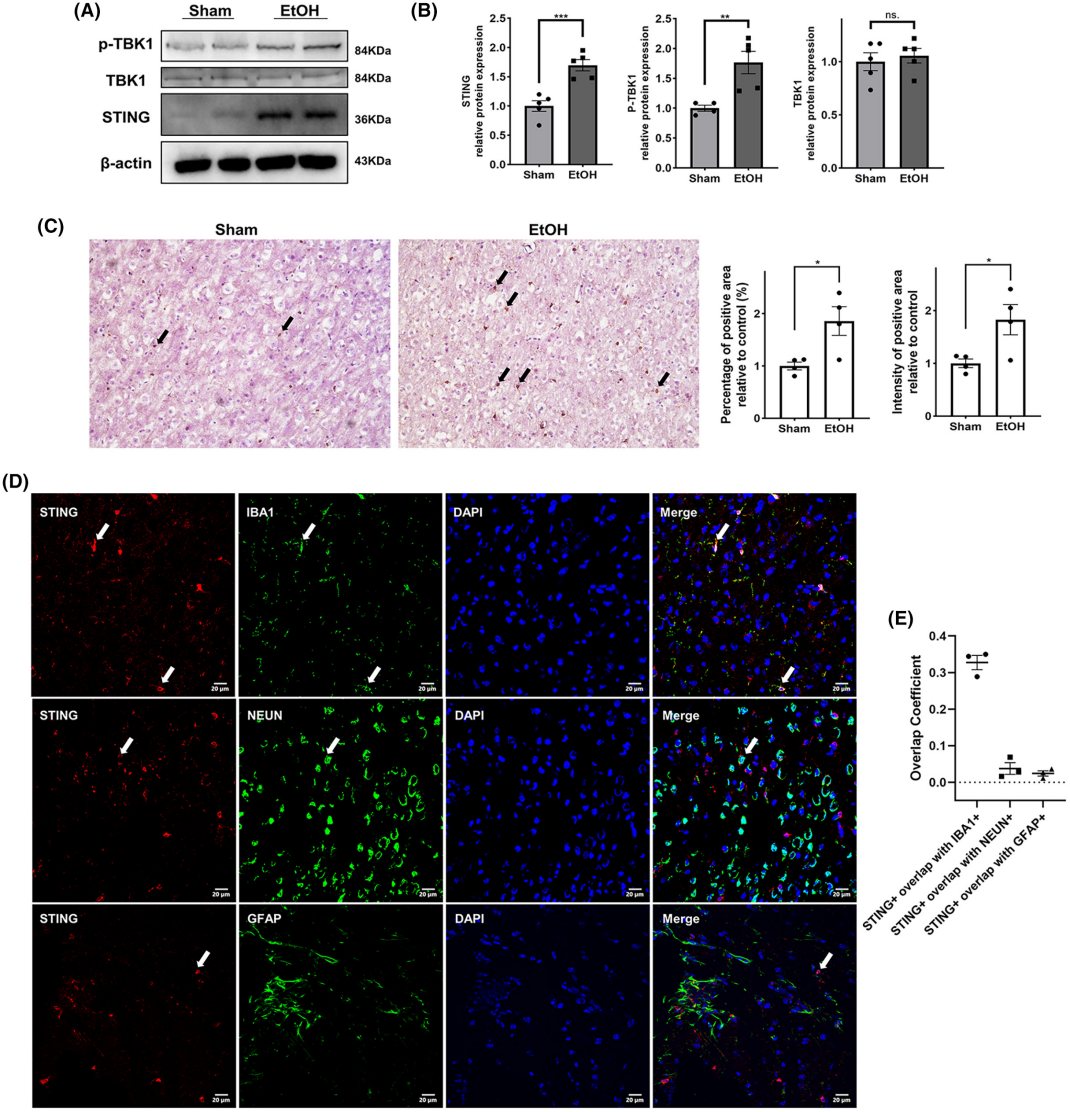
Expression of STING signaling pathway components in mice chronically exposed to ethanol. (A, B) Representative western blot images and quantitative analyses of the expression of STING signaling pathway components in the prefrontal cortex of mice treated with negative control treatment (Sham) or chronically exposed to alcohol (EtOH) (*n* = 4–5). (C) Representative IHC staining images and quantitative analyses of the expression of STING in the prefrontal cortex of mice in the sham group and EtOH group (original magnification 200×, *n* = 4). (D) Representative immunofluorescence double staining images and colocalization coefficients showing the colocalization of STING (red) with NeuN, GFAP, and IBA1 (green) in brain sections from mice chronically exposed to alcohol. *n* = 3. Scale bar = 20 μm. The data are expressed as the mean ± SEM; *p < 0.05, **p < 0.01, ***p < 0.001; ns, nonsignificant.

To examine whether STING signaling was activated resulting from chronic alcohol exposure‐induced dsDNA leakage, the expression patterns of genes associated with this pathway was assessed. In the mPFC of adolescent mice chronically exposed to alcohol, a significant increase in the protein expression of STING and the phosphorylation level of TBK1 was observed (Figure 2A,B). IHC staining also demonstrated significant upregulation of STING in the mPFC of alcohol‐treated mice in comparison to control mice (Figure 2C). We then investigated the distribution of STING in the mPFC of mice chronically exposed to alcohol. The positive expression of STING was localized to microglia and neurons but not astrocytes (Figure 2D,E). Based on these results, we focused our exploration of the role of STING signaling under alcohol exposure on microglia and neurons.

### 3.3 Activation of the STING signaling pathway increases the alcohol‐induced inflammatory response in BV2 cells

**FIGURE 3:**
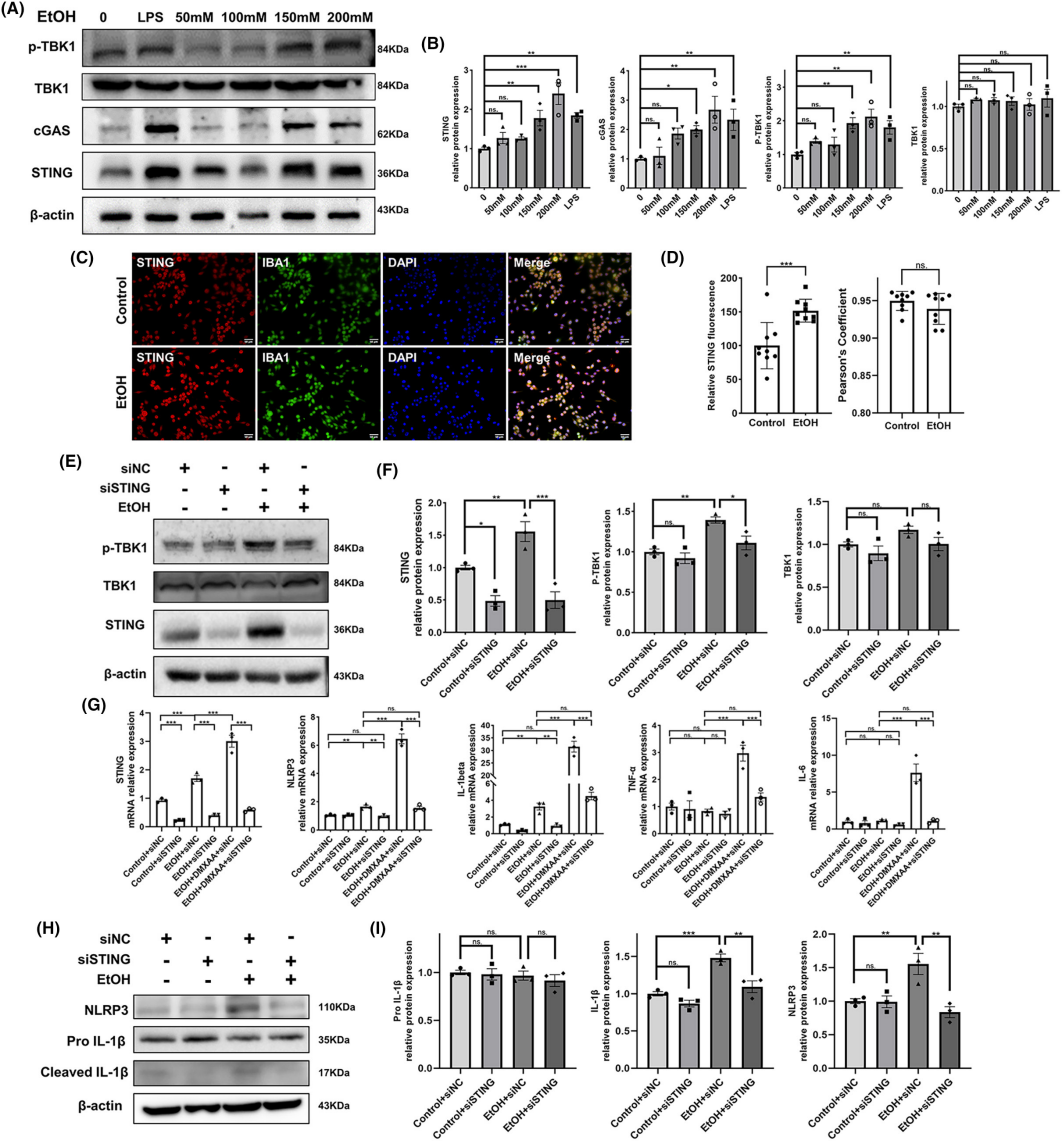
Expression of STING pathway components in microglia under alcohol exposure and the effect of STING on the inflammatory responses of microglia. (A, B) Representative immunoblot images and quantitative analyses of the expression of STING pathway‐associated genes in BV2 cells treated with different concentrations of alcohol and LPS (0.1 μg/mL) (*n* = 3). (C, D) Representative immunofluorescence staining images and quantitative analyses of STING expression in BV2 cells treated with control and EtOH (150 mM) (*n* = 9 random fields). Scale bar = 50 μm. (E, F) Representative images and quantitative analyses of STING, TBK1 and p‐TBK1 expression in the different groups (*n* = 3). Alcohol was administered 24 h after siRNA transfection. (G) Relative mRNA levels of STING and proinflammatory cytokines in BV2 cells given different treatments, as measured by qPCR (*n* = 3, DMXAA (10 μg/mL)). Alcohol and DMXAA were administered 24 h after siRNA transfection. (H, I) Representative immunoblot images and quantitative analyses of NLRP3 and IL‐1β expression in BV2 cells after different treatments (*n* = 3). BV2 cells were treated with alcohol 24 h after siRNA transfection. The data are expressed as the mean ± SEM; *p < 0.05, **p < 0.01, ***p < 0.001; ns, nonsignificant.

BV2 cells were exposed to lipopolysaccharide (LPS) and varying concentrations of alcohol for 24 h. A significant increase in the protein expression of STING, cGAS, and phospho‐TBK1 (p‐TBK1) in cells treated with LPS, 150 mM alcohol, and 200 mM alcohol was observed (Figure 3A,B). Immunofluorescence costaining of STING and IBA1 revealed that 150 mM alcohol treatment significantly increased the fluorescence intensity of STING, while it did not noticeably affect the Pearson's coefficient of STING and IBA1 in BV2 cells (Figure 3C,D). The mRNA level of STING was also found to be significantly elevated in BV2 cells treated with 150 mM alcohol compared with control cells (Figure 3G). Next, 150 mM alcohol was used for further experiments, and its impact on the immune response of BV2 cells and the involvement of STING in this response were investigated. The production of proinflammatory cytokines, including NLRP3 and IL‐1β, was significantly elevated in BV2 cells under alcohol exposure (Figure 3G–I). BV2 cells transfected with STING‐siRNA showed reduced mRNA and protein expression of STING, as determined by qPCR and western blotting (Figure S1). The elevated expression of STING and p‐TBK1 in BV2 cells induced by alcohol could be inhibited by STING‐siRNA transfection (Figure 3E,F). Moreover, alcohol‐induced increases in the mRNA levels of NLRP3 and IL‐1β in BV2 cells could be suppressed by STING‐siRNA transfection or exacerbated by DMXAA treatment. However, the mRNA levels of IL‐6 and TNF‐α in alcohol‐treated BV2 cells were not significantly different from those in control cells (Figure 3G). Additionally, the elevated protein levels of NLRP3 and cleaved IL‐1β due to alcohol exposure could be significantly inhibited through STING‐siRNA transfection in BV2 cells (Figure 3H,I).

### 3.4 Alcohol‐induced mitochondrial damage and activation of STING signaling in neurons

**FIGURE 4:**
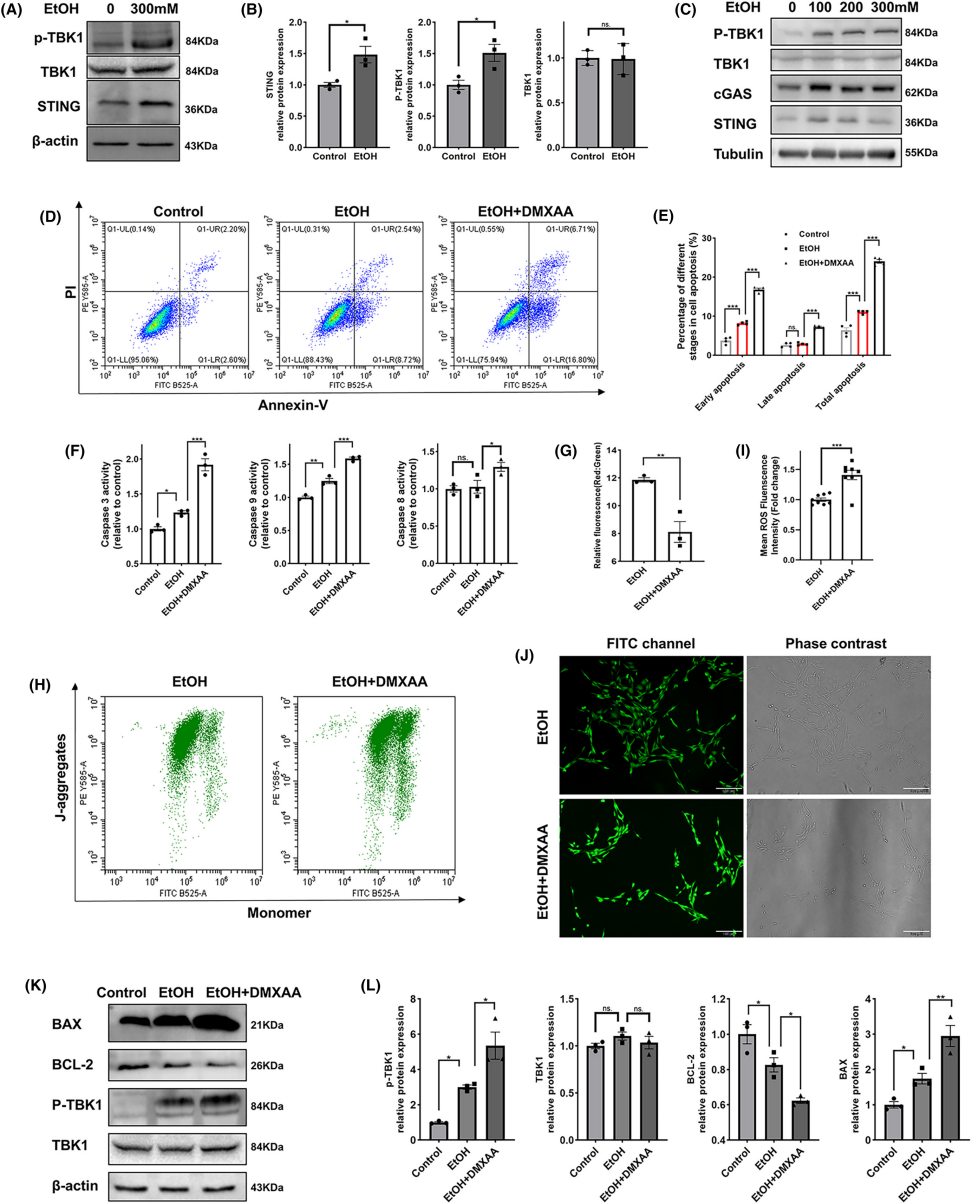
Activation of STING signaling induced by alcohol in neurons led to mitochondrial apoptosis. (A, B) Representative immunoblot images and quantitative analyses of the expression of STING pathway‐associated genes in PC12 cells treated with control and EtOH (300 mM) (*n* = 3). (C) Representative immunoblot images showing the expression of STING signaling pathway‐associated genes in primary neurons treated with various concentrations of alcohol (0, 100, 200, 300 mM). (D, E) Representative images and quantitative analyses of apoptotic PC12 cells in the different groups, as measured by flow cytometry (*n* = 4, DMXAA (50 μg/mL)). (F) Quantitative analyses of the activities of caspase 3, caspase 9, and caspase 8 in PC12 cells in the different groups (*n* = 3, DMXAA (50 μg/mL)). (G, I) Effects of DMXAA on the mitochondrial membrane potential of PC12 cells treated with alcohol, as measured by flow cytometry using JC‐1 (*n* = 3, DMXAA (50 μg/mL)). (H, J) Effects of DMXAA on ROS production in PC12 cells treated with alcohol, as measured by flow cytometry using JC‐1 (*n* = 3, DMXAA (50 μg/mL)). Scale bar = 100 μm. (K, L) Representative images and quantitative analyses of TBK1, p‐TBK1 and mitochondrial apoptosis‐associated gene expression in the different groups (*n* = 3, DMXAA (50 μg/mL)). The data are expressed as the mean ± SEM; *p < 0.05, **p < 0.01, ***p < 0.001; ns, nonsignificant.

PC12 cells and primary neurons were used to investigate STING signaling in neurons under alcohol exposure. The JC‐1 assays revealed that alcohol significantly decreased the mitochondrial membrane potential (Δψm) in PC12 cells, indicating that alcohol caused mitochondrial dysfunction in neurons (Figure S2A). Additionally, we observed an increased level of dsDNA in the cytosol and extracellular space in the alcohol‐exposed cells compared to the control cells, suggesting that alcohol‐induced damage to mitochondria led to the release of mtDNA (Figure S2B). Considering that the accumulation of dsDNA can activate the STING signaling pathway,^9^ we further examined cell‐intrinsic STING signaling in PC12 cells treated with alcohol. In alcohol‐treated PC12 cells, the protein expression levels of STING and p‐TBK1 were upregulated (Figure 4A,B). Next, we investigated whether the STING pathway activity could also be increased in primary neurons following alcohol exposure. The purity of the cultured primary neurons, as assessed by MAP2 and DAPI staining, was approximately 80% (Figure S3). Primary neurons were exposed to various concentrations of alcohol for 24 h, and increases in the protein expression of STING, cGAS, and p‐TBK1 were observed from the 100 mM alcohol treatment (Figure 4C).

### 3.5 Activation of STING signaling increases the alcohol‐induced mitochondrial apoptosis in PC12 cells

Given that STING has been linked to cell apoptosis,^7^, ^8^ we investigated whether the activation of STING signaling, triggered by alcohol, contributed to neuronal apoptosis. To assess this, we administered DMXAA to PC12 cells and observed upregulation of p‐TBK1 protein expression (Figure 4K,L). Flow cytometry confirmed that alcohol increased the early apoptosis rate of PC12 cells, and this effect was exacerbated by the addition of DMXAA (Figure 4D,E). Although DMXAA increased the late apoptosis rate of alcohol‐treated PC12 cells, the late apoptosis rate in the alcohol‐treated group was no significantly different from that in the control group (Figure 4D,E). We then investigated whether the extrinsic death receptor pathway or the intrinsic apoptotic pathway was involved in alcohol‐induced apoptosis in neurons. Our findings revealed that alcohol increased the activities of caspase 3 and caspase 9 in PC12 cells, and DMXAA further increased their activities (Figure 4F). There was no significant difference in the activity of caspase 8 (Figure 4F). Next, we investigated whether DMXAA contributed to mitochondrial dysfunction in PC12 cells. DMXAA exacerbated the alcohol‐induced decrease in the mitochondrial membrane potential (Figure 4G,I) and accumulation of ROS in PC12 cells (Figure 4H,J). The protein level of the proapoptotic molecule BAX was elevated by alcohol exposure and further increased by DMXAA in PC12 cells, while the protein level of the antiapoptotic molecule BCL2 was reduced (Figure 4K,L).

### 3.6 Activation of STING aggravates cognitive impairment in mice chronically exposed to alcohol

**FIGURE 5:**
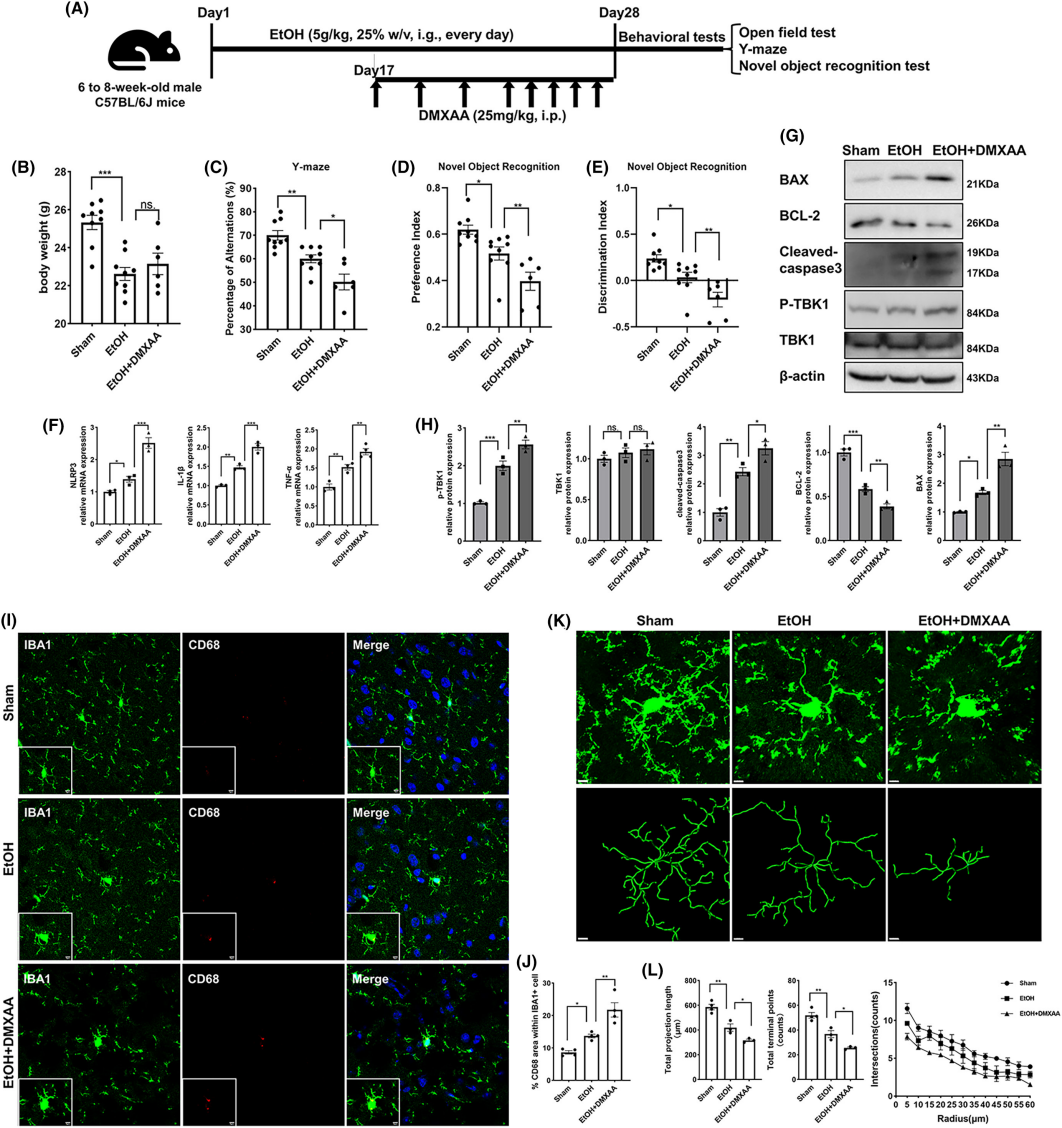
DMXAA aggravated chronic alcohol exposure‐induced cognitive impairment, neuroinflammation, apoptosis and microglial activation in mice. (A) Schematic diagram of animal experiment 1. (B) Body weights (g) of the different groups of mice at the end of treatment (*n* = 6–9). (C) Spontaneous alternation percentage of mice given different treatments in the Y maze test (*n* = 6–9). (D, E) Preference index and discrimination index of mice given different treatments in the novel object recognition test (*n* = 6–9). (F) Relative mRNA levels of proinflammatory cytokines in the prefrontal cortex of mice given different treatments (*n* = 3). (G, H) Representative immunoblot images and quantitative analyses of TBK1 expression and phosphorylation and mitochondrial apoptosis‐associated gene expression in the prefrontal cortex of mice given different treatments (*n* = 3). (I, J) Representative confocal images and quantitative analyses of CD68 (red) and IBA1 (green) co‐staining in brain sections of mice given different treatments (*n* = 4). Scale bar = 5 μm. (K) Representative images of microglial morphology in the different groups of mice visualized by immunofluorescence staining of IBA1 and skeletonization. Scale bar = 5 μm. (L) Quantitative analyses of the total length of projections, the total number of terminal points and the number of intersections of projections according to Sholl analysis of microglia in the different groups of mice (*n* = 3–4). The data are expressed as the mean ± SEM; *p < 0.05, **p < 0.01, ***p < 0.001; ns, nonsignificant.

Previous studies have demonstrated that chronic alcohol exposure induces anxiety and cognitive deficits in mice.^32^, ^33^ Herein, a chronic alcohol exposure model was employed and administered DMXAA to investigate the impact of STING activation on mice chronically exposed to alcohol. (Figure 5A). The body weight in the alcohol‐treated group was lower than that in the control group, while DMXAA did not affect body weight (Figure 5B). Open‐field tests revealed that mice subjected to chronic alcohol exposure exhibited reduced movement and increased fear when placed in a new open environment. This was evidenced by their decreased total distance traveled, less time spent in the central area, fewer total entries, and fewer entries into the central area. The administration of DMXAA did not significantly alter these alcohol‐induced behavioral changes (Figure S4B,C). The Y‐maze test was used to measure spatial working memory, and the novel object recognition test was used to test long‐term memory in mice. In comparison to control mice, alcohol‐treated mice made significantly fewer spontaneous alternations in the Y‐maze test, and DMXAA treatment further exacerbated this decrease (Figure 5C). In the novel object recognition test, the preference index and discrimination index decreased significantly in alcohol‐treated mice in comparison to control mice and further decreased in alcohol and DMXAA cotreated mice (Figure 5D,E).

### 3.7 Activation of STING signaling exacerbates neuroinflammation, apoptosis, and microglial activation in mice chronically exposed to alcohol

We examined whether the activation of STING could worsen neuroinflammation and apoptosis in vivo. The expression of p‐TBK1 was increased in the mPFC of mice after DMXAA treatment (Figure 5G). Mice exposed to alcohol exhibited higher levels of proinflammatory cytokines, including NLRP3, IL‐1β, and TNF‐α, in the mPFC than control mice (Figure 5F). Neuroinflammation in the mPFC was more severe in the alcohol and DMXAA cotreated group than in the alcohol‐treated group (Figure 5F). In addition, the expression of mitochondrial apoptosis‐associated genes changed in vivo. The protein levels of the proapoptotic molecule BAX and cleaved caspase 3 were increased by alcohol exposure, and this change was further aggravated by DMXAA (Figure 5G,H). Conversely, the protein level of the antiapoptotic molecule Bcl‐2 was decreased by chronic alcohol exposure and further decreased by DMXAA (Figure 5G,H).

Immunofluorescence staining of IBA1 and CD68 were used to assess the activation of microglia, as indicated by microglial morphology and microglial CD68 levels.^33^ The percentage of CD68‐positive area in IBA1‐positive cells in the mPFC was significantly higher in alcohol‐treated mice than in control mice, and this percentage was further increased in the alcohol group cotreated with DMXAA (Figure 5I,J). Changes in the morphology of microglia indicate alterations in their function.^33^ Microglia typically exhibit a ramified morphology, but upon activation, they display a deramified or amoeboid morphology. Herein, we found that chronic alcohol exposure led to the deramification of microglia with a decrease in the total length of projections, the total number of terminal points, and the number of intersections, as determined by Sholl analysis; these decreases were further aggravated after DMXAA treatment (Figure 5K,L).

### 3.8 Inhibition of STING signaling ameliorates cognitive impairment in mice chronically exposed to alcohol with modulation of neuroinflammation and apoptosis in the mPFC

**FIGURE 6:**
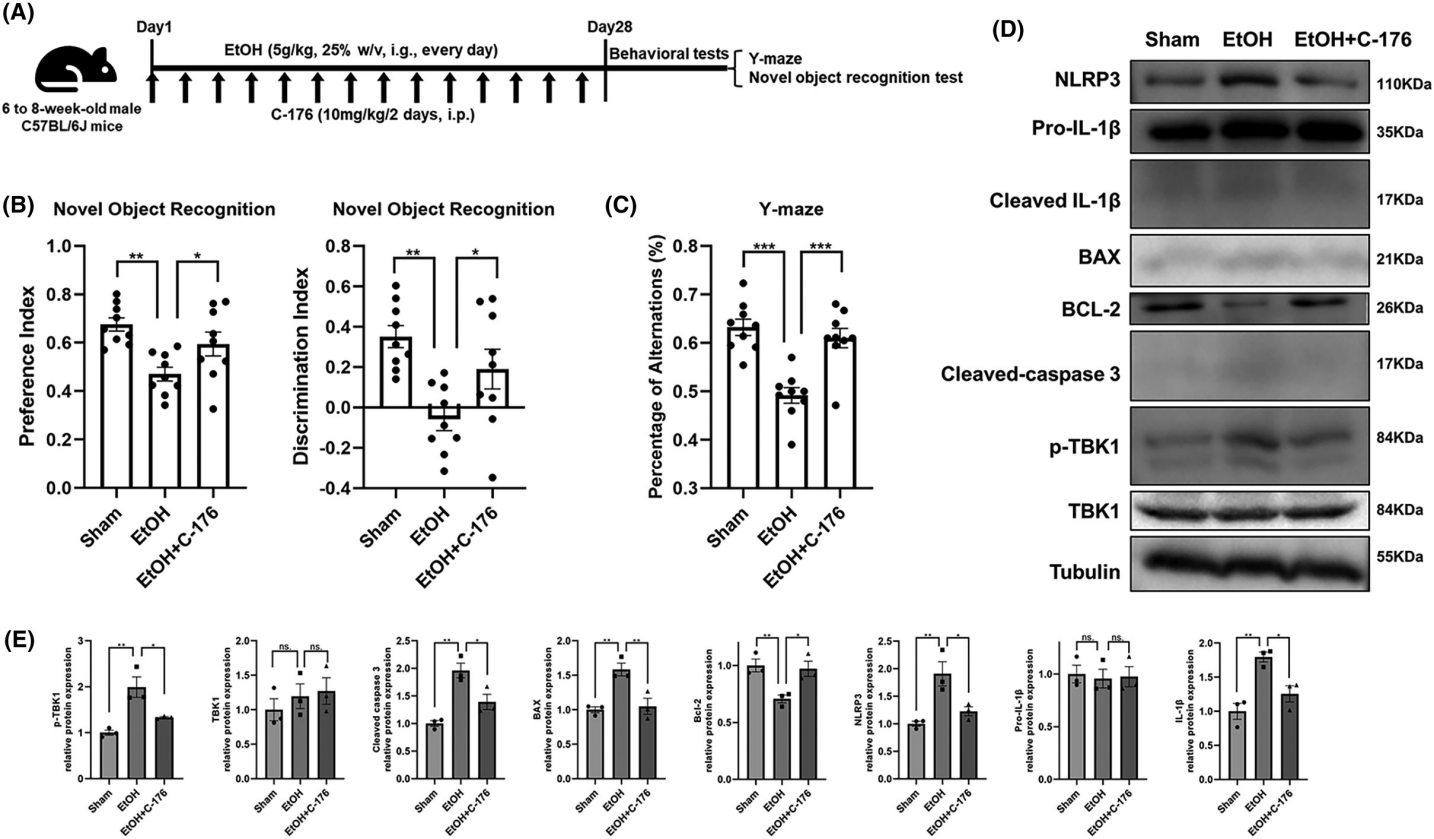
Administration of C‐176 ameliorated chronic alcohol exposure‐induced neuroinflammation and apoptosis in the prefrontal cortex and cognitive impairment in mice. (A) Schematic diagram of animal experiment 2. (B) Preference index and discrimination index of mice given different treatments in the novel object recognition test (*n* = 9). (C) Spontaneous alternation percentages of mice given different treatments in the Y maze test (*n* = 9). (D, E) Representative immunoblot images and quantitative analyses of TBK1 expression and phosphorylation, the expression of neuroinflammation‐associated genes including NLRP3 and IL‐1β, and the expression of mitochondrial apoptosis‐associated genes including cleaved caspase 3, BCL‐2, and BAX, in the prefrontal cortex of mice given different treatments (*n* = 3). The data are expressed as the mean ± SEM; *p < 0.05, **p < 0.01, ***p < 0.001; ns, nonsignificant.

To exclude the potential neurotoxicity of DMXAA, mice were administered with C‐176, a STING inhibitor, to investigate whether inhibition of STING signaling could ameliorate alcohol‐induced cognitive deficits (Figure 6A). The elevated expression of p‐TBK1 induced by chronic alcohol exposure could be effectively inhibited by C‐176 (Figure 6D,E). The cognitive decline induced by alcohol was reversed by C‐176 (Figure 6B,C). Specifically, C‐176 significantly reversed the reduced spontaneous alternation percentage in the Y‐maze test and the reduced preference index and discrimination index in the novel object recognition test. Mechanically, chronic alcohol exposure altered the protein expression of neuroinflammation‐associated genes (NLRP3 and cleaved IL‐1β) and mitochondrial apoptosis‐associated genes (BAX, BCL‐2, and cleaved caspase 3) in the mPFC. C‐176 reversed these changes, suggesting its potential to mitigate neuroinflammation and apoptosis induced by chronic alcohol exposure in the mPFC (Figure 6D,E).

## 4 DISCUSSION

The STING signaling pathway has been recognized as an essential pathologic mechanism of CNS diseases. Here, we showed that alcohol‐induced DNA damage as evidenced by increased circulating cell‐free mtDNA in individuals with AUD and mice chronically exposed to alcohol. We provided initial evidence that alcohol activated the STING in the mPFC, which led to neuroinflammation and mitochondrial apoptosis. Moreover, the STING agonist DMXAA aggravated alcohol‐induced cognition decline, while the STING inhibitor C‐176 exerted neuroprotection.

Preclinical studies have substantiated the occurrence of oxidative DNA damage in the CNS of mice as a result of alcohol consumption.^36^, ^37^ However, there is limited direct evidence for the occurrence of DNA damage in the brains of human patients with AUD. Under certain physiological or pathological conditions, such as pregnancy, organ transplantation, mitochondrial diseases, and cancers, affected tissues may release cell‐free DNA into the circulatory system throughout the body.^38^, ^39^ The quantification of circulating cell‐free DNA levels has also been used to evaluate CNS diseases such as schizophrenia and brain tumors.^40^, ^41^, ^42^ In the context of cancer, there is no connection between circulating cell‐free DNA levels and alcohol consumption.^43^ In the current study, we observed a significant rise in the levels of circulating cell‐free mtDNA in individuals with AUD and mice chronically exposed to alcohol compared with healthy control.

The involvement of STING signaling in various CNS diseases, such as ischemic stroke, Alzheimer's disease (AD), neonatal hypoxia‐ischemic encephalopathy (HIE), and traumatic brain injury (TBI), has been reported.^12^, ^44^, ^45^, ^46^, ^47^, ^48^ Activation of the STING has been confirmed in brain samples from patients with TBI and AD.^12^, ^45^ In this study, significant upregulated expression of genes related to the STING signaling pathway was observed in mice subjected to chronic alcohol exposure. In normal brain tissues, STING is mainly expressed in microglia and seldom expressed in astrocytes and neurons.^47^ However, under pathological conditions, elevated expression of STING could be induced in astrocytes and neurons. TBI induced activation of STING signaling in both neurons and astrocytes, with neuronal expression being restricted to the vicinity of the injury site and astrocytic STING expression being observed both ipsilateral and contralateral to the injury.^45^ After hypoxia‐ischemia, the expression of STING is significantly increased, and colocalization of STING with neuronal and microglial astrocytic markers is observed.^46^ In experimental subarachnoid hemorrhage (SAH), STING is mainly elevated in microglia.^47^ In our study, in the mPFC of mice chronically exposed to alcohol, STING was expressed in microglia and neurons. NEUN is a specific marker within the nucleus of neurons.^49^ While it has been reported that STING can be present in the nuclear lamina, STING is predominantly located in the cytosol.^50^ Therefore, the colocalization coefficient of NEUN and STING was low as shown in the above results.

Neuroinflammation, primarily mediated by microglial activation, is a crucial mechanism in CNS diseases, including AUD.^24^, ^51^ The STING, which is canonically involved in the innate immune response, has been implicated in neuroinflammation associated with CNS diseases.^44^ Inhibition of STING has been found to protect 5X FAD mice from amyloid‐β pathology and neuroinflammation.^12^ In cases of SAH, activation of microglial STING signaling promotes the polarization of microglia toward proinflammatory phenotypes and increases the generation of proinflammatory cytokines, such as NLRP3 inflammasome, IL‐1β, iNOS, IL‐6, TNF‐α, and MCP‐1.^47^ STING‐induced activation of PERK mediates IFN generation in TBI.^45^ In this study, activation of STING signaling contributed to alcohol‐induced microglial activation and generation of proinflammatory cytokines such as NLRP3 and IL‐1β.

In addition to its established role in facilitating the innate immune response, alternative functions of STING signaling, including its involvement in apoptosis, ferroptosis, and autophagy, have been reported.^6^, ^7^, ^8^ Prolonged activation of STING triggers mitochondria‐dependent apoptosis in B cells.^52^ A STING agonist, diABZI, induces the cleavage of caspase 3 and contributes to the development of acute respiratory distress syndrome (ARDS).^7^ Gain‐of‐function mutations in STING lead to increased susceptibility of T cells to apoptosis and the development of lung disease.^53^ In the context of HIE and SAH, STING activation leads to neuronal apoptosis.^46^, ^47^ Apoptosis is another key underlying mechanism of AUD.^22^, ^24^, ^54^ In our study, activation of STING signaling contributed to mitochondrial apoptosis both in vivo and in vitro. In PC12 cells, alcohol‐induced DNA damage activates the STING signaling pathway, which in turn exacerbates mitochondrial dysfunction and potentially increases the release of mtDNA. These findings suggest the existence of a potential positive feedback loop between the activation of STING signaling and DNA damage in alcohol‐treated cells.

It has been reported that the concentration for in vitro studies is recommended to be higher than that required to produce a similar effect in vivo.^55^, ^56^ Using a relatively high concentration of ethanol to better demonstrate its effects is permissible.^32^, ^55^, ^57^, ^58^ In addition, ethanol concentrations in the media in open vessels decreased over 60% in a 24‐h incubation period.^55^, ^59^ Limited by laboratory conditions, we could only use open containers. Therefore, we exposed cultured cells to alcohol concentrations ranging from 150 mM to 300 mM alcohol, surpassing the blood alcohol levels typically observed in individuals with AUD.

Previous studies have provided robust evidence supporting the pivotal role of neuroinflammation and apoptosis in alcohol‐induced cognitive impairment.^24^, ^32^ In the current study, DMXAA exacerbated alcohol‐induced neuroinflammation and apoptosis, whereas C‐176 provided relief from these effects. Furthermore, DMXAA resulted in poorer performance in the Y maze and novel object recognition tests, while C‐176 led to improved performance in these behavioral tests. These findings suggest that the involvement of STING signaling in the impact of chronic alcohol exposure on cognitive function may be linked, at least in part, to the induction of inflammation and apoptosis mediated by STING. Previous research has also highlighted the significance of inhibiting STING in regulating the interaction among neuroinflammation, apoptosis, and cognitive impairment. The STING inhibitor H‐151 ameliorates cognitive impairment in 5 × FAD mice.^12^ Similarly, C‐176 administrated to SAH model mice damaged the long‐term memory while CMA exacerbated cognitive impairments.^47^

Our study has several limitations. First, neuroinflammation induced by ethanol contributes to neurodegeneration, and microglia activated by alcohol exposure can eliminate neuronal synapses.^33^, ^60^, ^61^ The increased apoptosis observed in mice treated with alcohol and DMXAA may not have been only due to the cell‐intrinsic activation of STING signaling in neurons, but also may have been partly attributed to the activated STING signaling in microglia, subsequently inducing damage to neurons. Further investigation is necessary to examine the impact of the STING on microglia–neuron crosstalk in the context of alcohol exposure. Additionally, both the hippocampus and cortex are important regions that have well‐established roles in cognition^62^, ^63^ and are particularly sensitive to alcohol toxicity.^23^ Our study primarily focused on the mPFC, but of note, the hippocampus is also associated with alcohol‐induced cognitive impairment.^24^, ^32^ It is plausible that STING signaling can be triggered in the hippocampus of mice subjected to chronic alcohol exposure, and the effects of STING agonists or STING inhibitors on cognition are partially ascribed to alterations in neuroinflammation and apoptosis in the mPFC.

## 5 CONCLUSION

The present findings reveal the role of STING signaling in mediating alcohol‐induced cognition decline, neuroinflammation, and neuronal apoptosis; a graphical summary is provided in Figure S5. The levels of cell‐free mtDNA in plasma were elevated in human and mice following alcohol exposure. Activation of the STING contributed to alcohol‐induced cognitive impairment, mitochondrial apoptosis in neurons, and inflammatory response in microglia. This study suggests that targeting STING signaling may be a new potential therapeutic strategy for AUD.

## AUTHOR CONTRIBUTIONS

Xinrou Lin, Xiangpen Li, and Ying Peng designed the research. Xinrou Lin, Hongxuan Wang, Chenguang Li, and Lubin Zou conducted the experiments and analyzed the data. Xinrou Lin, Xiaoni Zhang. and Lei He wrote the initial draft of the manuscript. Xiangpen Li, Xiaoming Rong, and Ying Peng reviewed the manuscript and decided the final version. All authors contributed to the article and approved the submission of the manuscript.

## FUNDING INFORMATION

This work was supported by the Grant of China and Germany collaboration & exchange project (N0: M‐0746 to Y.P) and partly supported by the Grant of National Key R&D Program of China (2018YFC1314400, 2018 YFC13144‐01 to Y.P).

## CONFLICT OF INTEREST STATEMENT

None.

## CONSENT TO PARTICIPATE

The patient consent and approval were obtained from the Institutional Ethics Committee of Sun Yat‐Sen Memorial Hospital, Sun Yat‐Sen University.

## CONSENT FOR PUBLICATION

The authors agreed on the authorship and publication of this article.

## Supporting information


Fig. S1

Appendix S1

## Data Availability

The datasets used and/or analyzed during the current study are available from the corresponding author upon reasonable request.
