# Supplementary material for: Activation of STING signaling aggravates chronic alcohol exposure‐induced cognitive impairment by increasing neuroinflammation and mitochondrial apoptosis
Source: CNS Neurosci Ther. 2024 Mar 22;30(3):e14689. doi: 10.1111/cns.14689 (PMC10958405; doi:10.1111/cns.14689)
Supplement: Supplementary file 1 — Fig. S1 [file CNS-30-e14689-s001.docx]

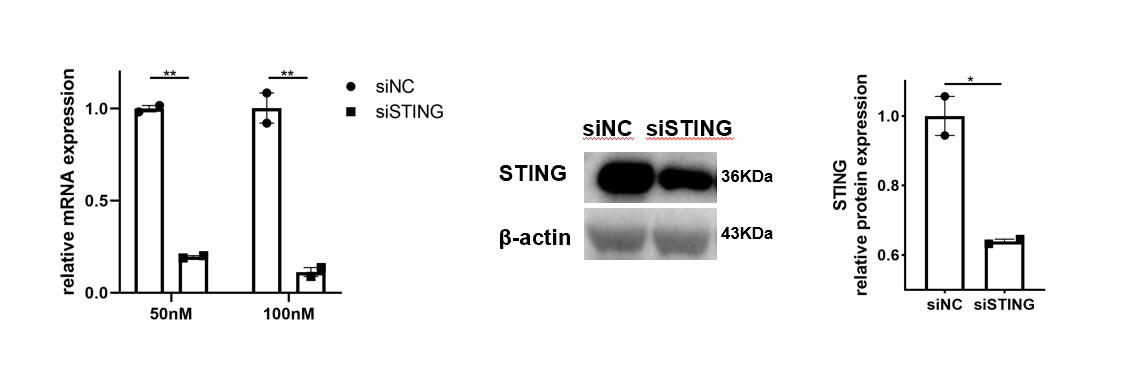


**Supplementary Figure 1. The transfection efficiency of STING-siRNA in BV2 cells, as measured by qPCR (50 nM or 100 nM siRNA) and western blot (50 nM siRNA).**


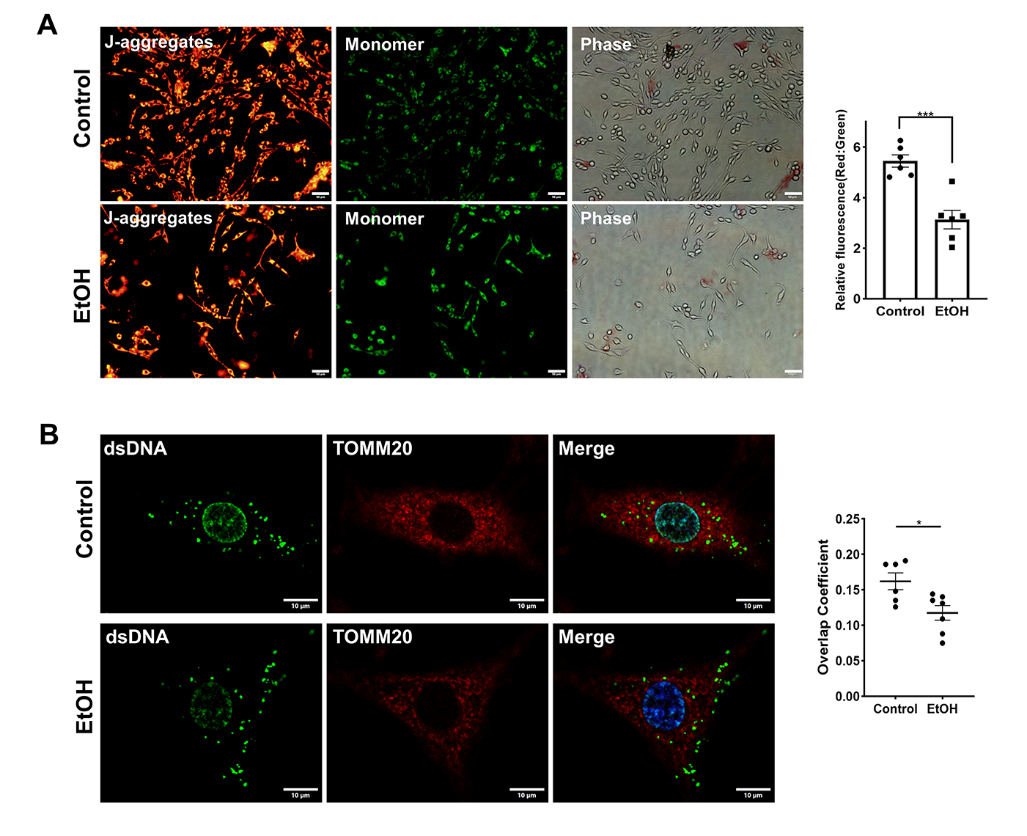


**Supplementary Figure 2. Alcohol caused mitochondrial dysfunction in PC12 cells. A** Representative images and quantitative analyses of the mitochondrial membrane potential, as measured using JC-1, in PC12 cells treated with control or EtOH (300 mM) (n=6 random fields). Scale bar = 50 μm. **B** Representative confocal images and quantitative analyses of colocalization coefficients of dsDNA and TOMM20 in PC12 cells treated with control or EtOH (300 mM). (n=6-7 random fields). Scale bar = 10 μm.


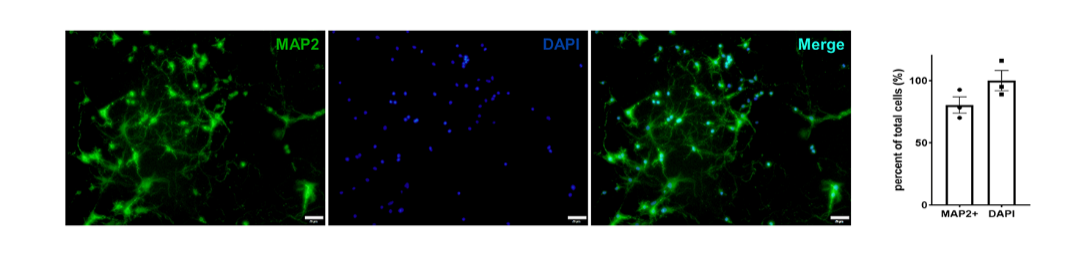


**Supplementary Figure 3.** Representative images of cultured primary neurons and quantitative analysis of their purity. Scale bar = 50 μm.


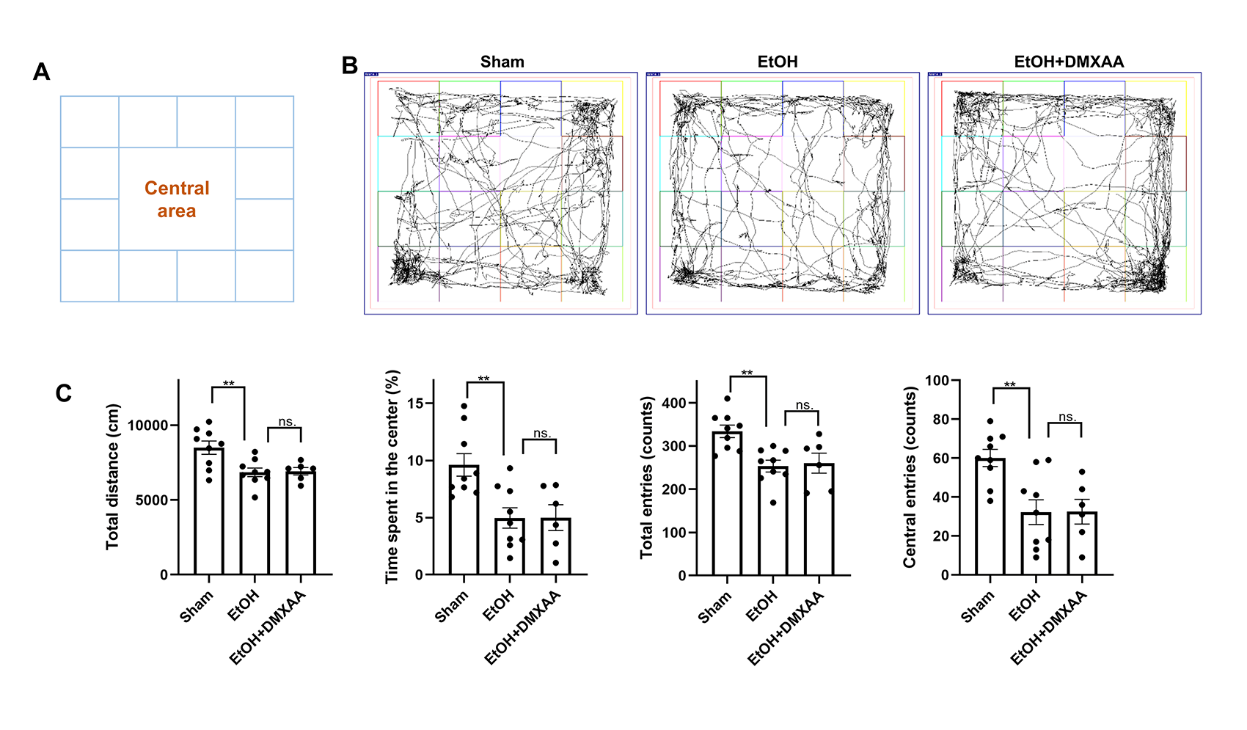


**Supplementary Figure 4. Results of the open field test. A** Schematic diagram of the central area. **B** Representative tracks of mice given different treatments. **C** Quantitative analyses of the performance of mice given different treatments (n=6-9) in the open field test.


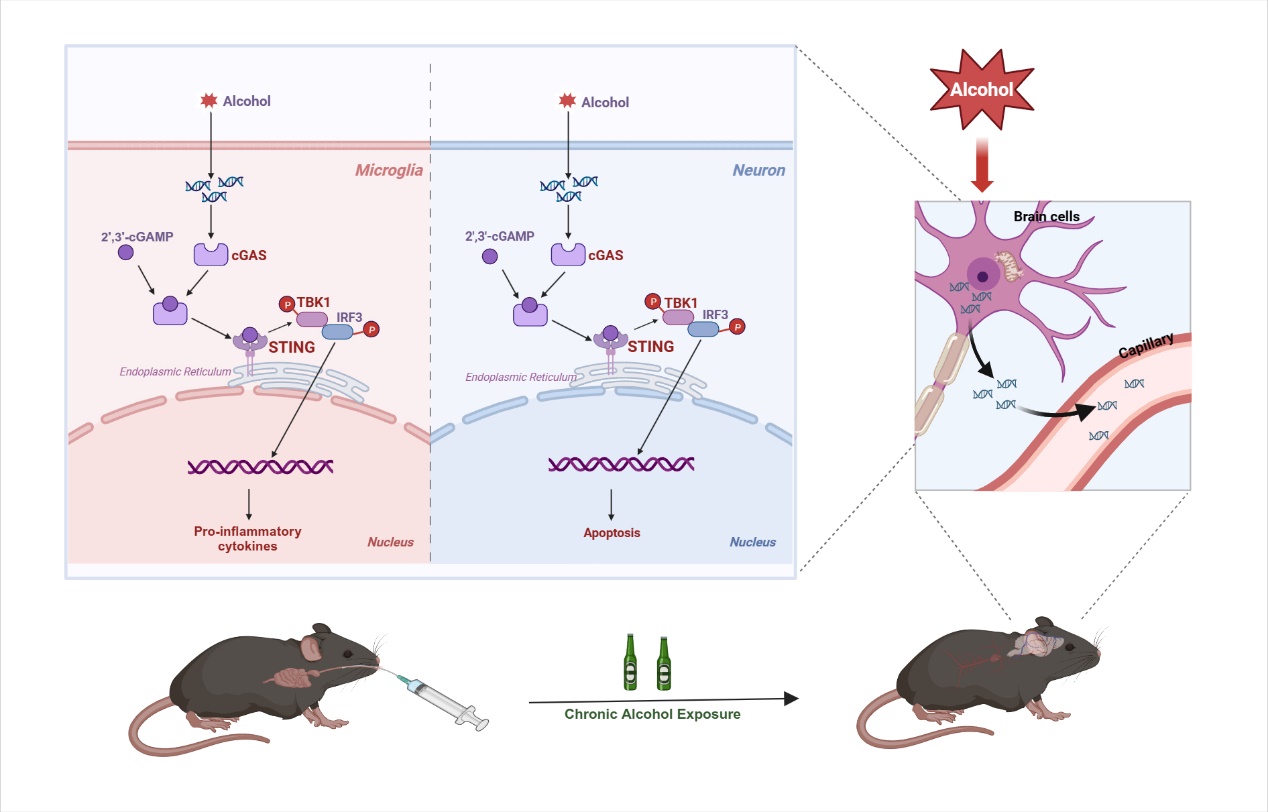


**Supplementary Figure 5. Graphical summary depicting DNA damage induced by chronic alcohol exposure and subsequent activation of the STING signaling pathway.** Under conditions of chronic alcohol exposure, brain cells are damaged and aberrantly release dsDNA into both the cytosol and extracellular space. Extracellular dsDNA enters the circulatory system so that cell-free mtDNA can be detected in plasma. Simultaneously, cytosolic dsDNA triggers the activation of the STING signaling pathway in microglia and neurons. The activated STING signaling pathway in microglia contributes to alcohol-induced neuroinflammation, while its activation in neurons is implicated in alcohol-induced apoptosis.

**Supplementary Table 1. The primer sequences of targeted genes**

| **Target gene** | **Primer forward sequences** | **Primer reverse sequences** |
| --- | --- | --- |
| mouse STING | CATGTCCAGTCCAGTTGGATGTT | TCAACCGCAAGTACCCAATGT |
| mouse NLRP3 | ATTTGTACCCAAGGCTGCTATC | CAACGGACACTCGTCATCTTC |
| mouse TNF-α | GAGAAGGGGGACCAACTCAG | ACCCATTCCCTTCACAGAGC |
| mouse IL-1β | GAAATGCCACCTTTTGACAGTG | TGGATGCTCTCATCAGGACAG |
| mouse IL-6 | TCTATACCACTTCACAAGTCGGA | GAATTGCCATTGCACAACTCTTT |
| mouse β-actin | CTAAGGCCAACCGTGAAAAG | ACCAGAGGCATACAGGGACA |
| mouse GAPDH | ACTTCAACAGCAACTCCCACT | TCTCTTGCTCAGTGTCCTTGC |
| mouse mt-COI | GCCCCAGATATAGCATTCCC | GTTCATCCTGTTCCTGCTCC |
| mouse mt-Dloop | GCCCATGATCAACATAACTG | CCTTGACGGCTATGTTGATG |
| mouse 18S | TAGAGGGACAAGTGGCGTTC | CGCTGAGCCAGTCAGTGT |
| rat TNF-α | GCATGATCCGAGATGTGGAACTGG | CGCCACGAGCAGGAATGAGAAG |
| rat β-actin | CGAGTACAACCTTCTTGCAGC | ACCCATACCCACCATCACAC |
| human MT-ND1 | CCCTAAAACCCGCCACATCT | GAGCGATGGTGAGAGCTAAGGT |
| human MT-ND6 | CCAATCCTACCTCCATCGCT | GAGTATCCTGAGGCATGGGG |
| human GAPDH | GTATTCCCCCAGGTTTACATGTTC | ACTCACTCCTGGAAGATGGTGAT |
| human TERT | CAATGCCTCACATAAATGCTACC | AGTGCAAAGCTTCTGTCTCCTTCT |

**Supplementary Table 2. Demographics and clinical characteristics of healthy controls and patients with AUDs in this study**

|  | **Sex** | **Age, years** | **Duration of drinking, years** |
| --- | --- | --- | --- |
| Healthy controls | Male | 46.9 ± 6.1 | *NA* |
| AUDs | Male | 49.1 ± 7.52 | 16.54 ± 9.67 |

AUDs, alcohol use disorders.

*NA* not applicable

**Supplementary Table 3. Information about antibodies used in this study.**

| **Antibody** | **Dilution** | **Source** | **Identifier** |
| --- | --- | --- | --- |
| Rabbit anti-STING | 1:1000(WB)  1:200 (IF)  1:500 (IHC) | Proteintech | 19851-1-AP |
| Rabbit anti-cGAS | 1:1000(WB) | Cell Signaling Technology | 31659S |
| Rabbit anti-TBK1 | 1:1000(WB) | Proteintech | 28397-1-AP |
| Rabbit anti-p-TBK1 | 1:1000(WB) | Abcam | Ab109272 |
| Rabbit anti-BAX | 1:1000(WB) | Proteintech | 50599-2-Ig |
| Rabbit anti-Bcl-2 | 1:1000(WB) | Beyotime | AF6285 |
| Rabbit anti-cleaved caspase 3 | 1:1000(WB) | Proteintech | 19677-1-AP |
| Mouse anti-NeuN | 1:200 (IF) | Proteintech | 66836-1-Ig |
| Mouse anti-GFAP | 1:200 (IF) | Beyotime | AF0156 |
| Mouse anti-IBA1 | 1:200 (IF) | Abcam | Ab283319 |
| Rabbit anti-CD68 | 1:500 (IF) | Cell Signaling Technology | 97778S |
| Rabbit anti-MAP2 | 1:200 (IF) | Proteintech | 17490-1-AP |
| Rabbit anti-TOMM20 | 1:200 (IF) | Proteintech | 11802-1-AP |
| Mouse anti-dsDNA | 1:200 (IF) | Millipore | CBL186 |
| Rabbit anti-α-tubulin | 1:2000(WB) | Proteintech | HRP-66031 |
| Mouse anti-β-actin | 1:2000(WB) | Proteintech | HRP-60008 |
| HRP-conjugated goat anti-rabbit secondary antibody | 1:5000(WB) | Proteintech | SA00001-2 |
| HRP-conjugated goat anti-rabbit secondary antibody | 1:5000(WB) | Proteintech | SA00001-1 |
| Alexa Fluor 488-labeled goat anti-rabbit secondary antibody | 1:200 (IF) | Beyotime | A0423 |
| Alexa Fluor 488-labeled goat anti-mouse secondary antibody | 1:200 (IF) | Beyotime | A0428 |
| Alexa Fluor cy3-labeled goat anti-rabbit secondary antibody | 1:200 (IF) | Beyotime | A0516 |
| Alexa Fluor cy3-labeled goat anti-mouse secondary antibody | 1:200 (IF) | Beyotime | A0521 |
