# Supplementary material for: Activation of STING signaling aggravates chronic alcohol exposure‐induced cognitive impairment by increasing neuroinflammation and mitochondrial apoptosis
Source: CNS Neurosci Ther. 2024 Mar 22;30(3):e14689. doi: 10.1111/cns.14689 (PMC10958405; doi:10.1111/cns.14689)
Supplement: Supplementary file 2 — Appendix S1 [file CNS-30-e14689-s002.docx]

**Supplementary materials and methods**

**Behavioral tests**

**Open field test**

The open field test was performed to evaluate the locomotor activity and anxiety of mice. The mice were placed into the center of an open opaque acrylic box (50 x 50 x 40 cm) divided into 16 zones and allowed to move freely for 10 minutes. The behaviors of the mice were recorded and analyzed using a video tracking system (Smart v3.0.06, Panlab Harvard Apparatus, Barcelona, ES). The central four zones were assigned as the central area (Fig. S4A). The total distance traveled, the time spent in the central area, the total number of entries, and the number of entries into the central area were measured. The chambers were cleaned with 75% ethanol before every trial.

**Novel object recognition test**

Novel object recognition tests were carried out in a black opaque acrylic box (50 x 50 x 40 cm) to evaluate the working memory. On the first day, the mice were allowed to move freely for 10 minutes. On the second day, the mice were allowed to explore two identical objects (A+A) for 10 minutes. On the third day, one of the objects was substituted with a new object (B) that had a different size, shape, and color. The preference index (PI) was calculated as the time spent exploring the novel object divided by the total time spent exploring both novel and familiar objects (PI = B / (A+B) x 100%). The discrimination index (DI) was determined by subtracting the time spent exploring the familiar object from the time spent exploring the novel object, and then dividing that difference by the total exploration time (DI = [(B-A) / (A+B)] x 100%). The objects and chambers were cleaned with 75% ethanol before every trial.

**Y-maze**

A Y-maze apparatus (30 x 8 x 15 cm), which had three arms (1, 2, and 3) positioned at a 120° angle from each other, was employed to evaluate spatial working memory. Each mouse was given 10 minutes to freely explore the maze. Spontaneous alternations were determined as three consecutive entries into different arms, such as 123, 132, 231, 213, 312 and 321. Spontaneous alternation percentage was calculated as the number of spontaneous alternations / (the total number of entries - 2) x 100%. The maze was cleaned with 75% ethanol before every trial.

**Primary neuronal culture**

Primary neurons were obtained from C57BL/6J mouse pups on postnatal day 0-1 as previously described[1,2]. The cortex and hippocampus were dissected out in cold HBSS and digested in 0.125% trypsin at 37°C for 15 minutes. The cells were centrifugated at 1000 × g for 5 minutes and resuspended in DMEM supplemented with 10% FBS. Four hours later, the cultured medium was changed into neurobasal-A medium (10888022, Gibco) supplemented with B-27 (17504044, Gibco) and GlutaMAX (35050061, Gibco). After 7 DIV (days in vitro) in culture, the primary neurons were used for further experiments.

**RNA extraction and qPCR**

RNA was isolated using TRIzol reagent (Vazyme, China) and subsequently reverse transcribed into complementary DNA (cDNA) using a PrimeScript RT Reagent Kit (Vazyme, China) following the manufacturer’s guidelines. The resulting cDNA was employed for quantitative real-time polymerase chain reaction (RT‒qPCR) using SYBR Green reaction Mix (Vazyme, China) and a LightCycler 384 System (Roche, Basel, Switzerland). Relative mRNA expression levels were calculated using the 2-ΔΔCt method, with GAPDH or β-actin serving as the internal control. The sequences of primers for the target genes are shown in Supplemental Table 1.

**Western blotting**

RIPA buffer supplemented with phosphatase and protease inhibitors (Beyotime, China) was used to homogenize the cells or brain tissues. Total proteins (40 μg) were separated by 10% or 12% SDS‒PAGE and transferred to PVDF membranes. Next, the membranes were blocked with 5% milk at room temperature for 1 hour and incubated overnight at 4°C with primary antibodies. Following washing with TBST three times, the membranes were incubated with secondary antibodies at room temperature for 2 hours. The information about antibodies used in this experiment are listed in Supplemental Table 3. β-Actin and α-Tubulin were used as the loading controls. The densities of the immunoreactive protein bands were measured using Fiji software.

**Apoptosis assay**

PC12 cells were stained with propidium iodide (PI) and FITC Annexin V at room temperature in the dark for 15 minutes based on the manufacturer’s instructions (Yeasen, 40305ES60). The apoptosis rate of cells under various treatment conditions was determined using a flow cytometer (Beckman Coulter, CytoFLEX, USA).

**Immunofluorescence (IF)**

After fixation in ice-cold 4% paraformaldehyde and dehydration in a sucrose gradient ranging from 10% to 30%, the brains were sliced into 20-µm-thick sections. Following blocking with 5% normal goat serum, the sections were incubated with primary antibodies overnight at 4°C. Subsequently, the sections were washed with PBS three times and incubated with secondary antibodies at room temperature for 2 hours. The specific antibodies used in this experiment can be found in Supplemental Table 3. A confocal laser scanning microscope (FV30S-SW, Olympus) was employed to capture immunofluorescence images. At least 4 randomly selected fields from each animal were analyzed using Fiji software.

For the analysis of microglial morphology, images of sections stained with IBA1 were captured using the z-stack function of an Olympus FV3000 confocal microscope at a magnification of 40X. Each image consisted of 15-25 slices at a fixed interval. Three to six randomly selected fields from each animal were used for subsequent analysis by Imaris v9.0 software (Bitplane, Switzerland).

**Immunohistochemistry (IHC)**

Mouse brain sections were blocked with endogenous peroxidase at room temperature for 10 minutes and 5% normal goat serum at room temperature for 1 hour. The sections were then incubated with an anti-STING primary antibody overnight at 4°C and subsequently with a biotinylated secondary antibody at room temperature for 30 minutes. Finally, the sections were stained with DAB chromogen and hematoxylin, dehydrated and sealed with neutral resin. Images of 6 randomly selected fields from each animal were analyzed by Fiji software.

**Immunocytochemistry (ICC)**

The cells were cultured on glass coverslips and fixed with 4% paraformaldehyde. The cells were permeabilized with 0.1% Triton™ X-100 in PBS for 20 minutes and blocked with 5% normal goat serum at room temperature for one hour. Subsequently, the cells were incubated with primary antibodies overnight at 4°C and with secondary antibodies at room temperature for 1 hour. A confocal laser scanning microscope (FV30S-SW, Olympus) was employed to capture immunofluorescence images. Fiji software was utilized to analyze at least 6 randomly selected fields from three independent experiments per group.

**Measurement of the mitochondrial membrane potential (Δψm)**

A JC-1 (5,5′,6,6′-tetrachloro-1,1′,3,3′-tetraethylbenzimidazo lcarbocyanine iodide) assay kit (C2006, Beyotime) was employed to measure the ΔΨm according to the manufacturer’s instructions. Briefly, PC12 cells were treated with 10 μg/ml JC-1 for 30 min at 37°C and analyzed using a flow cytometry (Beckman Coulter, CytoFLEX, USA) or an inverted fluorescence microscope (Olympus IX73). The relative ΔΨm was calculated by dividing the red fluorescence intensity (JC-1 aggregates) by the green fluorescence intensity (JC-1 monomers). A decrease in this ratio indicated mitochondrial dysfunction[3].

**Reactive oxygen species (ROS) measurement**

ROS generation was measured using 2′,7′-dichlorofluorescein diacetate (DCFH-DA; S0033S, Beyotime, China) based on the manufacturer’s instructions. In brief, PC12 cells were first washed with PBS and then treated with DMEM containing DCFH-DA (10 μM) at 37°C in the dark for 30 min. Next, the cells were washed with DMEM three times and visualized by an inverted fluorescence microscope (Olympus IX73).

**Activities of caspase 3, caspase 8 and caspase 9**

The activities of caspase-3, caspase-8, and caspase-9 were assessed using commercially available assay kits (C1116, C1152, and C1158, Beyotime, China) following the manufacturer’s instructions. Briefly, cells were treated with lysis buffer, harvested and centrifuged at 16,000× g for 15 minutes. The resulting supernatants were collected and incubated with specific substrates, namely, Ac-DEVD-pNA (acetyl-Asp-Glu-Val-Asp p-nitroanilide), Ac-IETD-pNA (acetyl-Ile-Glu-Thr-Aspp-nitroanilide) and Ac-LEHD-pNA (acetyl-Leu-Glu-His-Asp p-nitroanilide). Enzymatic activity was determined by measuring the absorbance at 405 nm and using a pNA (p-nitroanilide) standard curve. To account for variations in protein content, the absorbance values were normalized to the total amount of protein quantified using the Bradford method.

1. Beaudoin GM, 3rd, Lee SH, Singh D, et al. Culturing pyramidal neurons from the early postnatal mouse hippocampus and cortex. Nature protocols. 2012;7(9):1741-54. doi: 10.1038/nprot.2012.099.

2. Ryou MG, Choudhury GR, Li W, et al. Methylene blue-induced neuronal protective mechanism against hypoxia-reoxygenation stress. Neuroscience. 2015;301:193-203. doi: 10.1016/j.neuroscience.2015.05.064.

3. Nan B, Zhao Z, Jiang K, et al. Astaxanthine attenuates cisplatin ototoxicity in vitro and protects against cisplatin-induced hearing loss in vivo. Acta pharmaceutica Sinica B. 2022;12(1):167-81. doi: 10.1016/j.apsb.2021.07.002.
